# Heterogeneity and other problems in a pooled analysis of snus use and mortality

**DOI:** 10.12688/f1000research.52127.1

**Published:** 2021-05-14

**Authors:** Brad Rodu, Nantaporn Plurphanswat

**Affiliations:** 1 James Graham Brown Cancer Center, University of Louisville, Louisville, KY, 40202, USA; 2 Department of Medicine, School of Medicine, University of Louisville, Louisville, KY, 40202, USA

**Keywords:** snus use, smokeless tobacco, mortality follow-up study, Swedish Collaboration on Health Effects of Snus Use, heterogeneity

## Abstract

A recent analysis of Swedish snus use and mortality combined eight Swedish datasets and found that exclusive Swedish male snus users have statistically significant increased mortality from all causes, cardiovascular diseases and other causes. These findings, from the Swedish Collaboration on Health Effects of Snus Use, are in sharp contrast with previous pooled results from the same group. The discrepant results may be indicative of unresolved statistical problems that haven’t been addressed by the collaboration authors in any of their studies.

The most important problem is unresolved heterogeneity among the eight cohorts, which we describe in detail, and we show how the use of the random effects method by the authors was not sufficient. We explain why the tables in the article are uninformative, and we demonstrate why the exclusion of smokers in the analysis was not validated and eliminated important information. Finally, we strongly recommend some straightforward and easily implemented corrective measures.

## Introduction

A recent analysis by Byhamre *et al.* of Swedish snus use and mortality combined eight Swedish datasets and found that exclusive Swedish male snus users have statistically significant increased mortality from all causes (adjusted hazard ratio, aHR = 1.28, 95% confidence interval, CI = 1.20 – 1.35), cardiovascular diseases (aHR = 1.27, CI = 1.15 – 1.41) and other causes (aHR = 1.37, CI = 1.24 – 1.52), as well as a 12% elevation in cancer mortality (aHR = 1.12, CI = 1.00 – 1.26). ^1^ These findings, from the Swedish Collaboration on Health Effects of Snus Use, are in sharp contrast with previous pooled results from the same group that found no significant increases in incidence and/or mortality from acute myocardial infarction, ^2^ stroke, ^3^ atrial fibrillation, ^4^ pancreatic cancer, ^5^ oral cancer ^6^ and colorectal cancer. ^7^ The latter study did report a significant association of exclusive current snus use and rectal cancer (aHR = 1.38, CI = 1.07 – 1.77). ^7^

We are concerned that the discrepant results between the latest analysis and previous published studies may be indicative of unresolved statistical problems that haven’t been addressed by the collaboration authors in any of their studies. These problems include unresolved heterogeneity among the eight cohorts, for which the random effects method the authors used was not a sufficient remedy. In addition, the tables in the article are uninformative, and the exclusion of smokers in the analysis was not validated and eliminated important information. There are straightforward and easily implemented corrective measures that the authors could undertake.

## The pooled cohorts are highly heterogeneous

First, the cohorts in the study are heterogeneous, as our Table 1 demonstrates. The eight cohorts were all described as “population-based,” but only four fit that description, and two of those were duplicative. The population-based cohorts were from: (a) the most populous Swedish County – the Stockholm Public Health Cohort (StPHC); (b) a Diet and Cancer Study in the third most populous city, Malmö; (MDCS) (c) the Scania Public Health Cohort (ScPHC), which contains Malmö; and (d) the Multinational Monitoring of Trends and Determinants in Cardiovascular Disease (MONICA) cohort, from rural Västerbotten and Norbotten Counties, which make up 34% of Sweden’s land area but only 5% of its population. The other four cohorts were convenience samples (the first three are from throughout Sweden). They consisted of: (a) the construction worker cohort (CWC); (b) participants in a charity walk, the National March Cohort (NMC); (c) twins born from 1926 to 1958 (Screening Across the Lifespan Twin, or SALT, Study); and (d) employees in three counties (Work, Lipids and Fibrinogen, WOLF study), which included Stockholm.

**Table 1.** Characteristics of the pooled Swedish cohorts.

Cohort Type and Name	Enrollment years	End of follow-up	Study population source	Number of Male Participants	Mean age at enrollment in years (range)	Number of Deaths	Mean age at death in years	Current smokers (%)	Current snus users (%)
**Population-based**									
Stockholm Public Health Cohort (StPHC)	2002, 2006, and 2010	2011	Stockholm	39406	50 2002, 2006 (18-84) 2010 (18)	1465	76	13	18
Malmö Diet and Cancer Study (MDCS)	1991-1996	2013	Malmö	12120	59 (45-73)	4372	75	27	7
Scania Public Health Cohort (ScPHC)	1999-2000	2008	Scania	6201	48 (18-80)	231	76	21	20
Multinational Monitoring of Trends and Determinants in Cardiovascular Disease (MONICA)	1986, 1990, 1994, 1999, 2004	2008	Norrbotten and Västerbotten	4563	48 1986,1990: (25-64) 1994,1999, 2004 (25-74)	643	70	22	24
**Convenience**									
Construction Worker Cohort (CWC)	1978-1993	2004	Blue/white collar construction workers	279897	34	31,429	66	46	27
National March Cohort (NMC)	1997	2010	Participants in a charity walk	15318	52 (18-94)	2,531	78	7	9
Screening Across the Lifespan Twin Study (SALT)	1998-2002	2010	Twins born 1926-1958	18331	56 (40-76)	2522	71	17	16
Work, Lipids and Fibrinogen Study (WOLF)	1992-1997	2009	Employees from 60 companies Stockholm, Västernorrland, Jämtland.	7189	42 (18-64)	265	61	20	23

The heterogeneity of the samples is further illustrated by other characteristics described in Table 1, including the mean age at death. It was 61 years among employees, which was much lower compared not only with the other cohorts, but with men in Sweden during the same recruitment and follow-up periods. ^8^ Other diverse variables were the prevalence of smoking and snus use. As we discuss in detail later, Byhamre *et al.* ^1^ excluded smokers from their analysis, but didn’t justify their choice, which is not innocuous and contributes to the estimates that the authors conclusions are based upon. They also, misleadingly, included these omitted observations in their table describing their cohorts. So, we point out that smoking and snus use prevalence varied from very low (7% and 9%, respectively) among charity walkers to very high (46% and 27%) among construction workers.

The construction workers account for 72-73% of both the sample and the number of deaths. Their mean enrollment age was 34 years, which was vastly different than four other cohorts that recruited men aged 50+ years. The first mortality follow-up study of snus use involving this cohort, published in 1994, ^9^ had an unusual and unresolved distribution of excess deaths. ^10^

Pooled analyses are intended to yield results that are generalizable to the pool. However, the vast differences in the characteristics of the cohorts that have been disclosed raise a concern whether the findings are interpretable as population estimates. As discussed, only four cohorts were from population-based samples, and three enrolled very small numbers. The participant selection process in the vastly larger convenience sample cohorts is the dominant influence of this study’s results and, given that the employee cohort is by far the largest and makes up a large majority of the pooled sample, the very different snus use and mortality in this cohort is reason alone to question the external validity of the results even if they are correct, which we do not think is the case.

## The methodology was flawed

Byhamre *et al.* ^1^ used a random-effects method to account for the variation in mortality across cohorts, in which they assumed that there is no correlation between individual cohorts and snus use. However, that method also assumes that unmeasured factors specific to a cohort are unrelated to either snus use or mortality, which is almost surely unlikely to hold in practice. For example, as we show in Table 1, the mean age at enrollment in the four population-based cohorts was 48 to 59 years, compared with 34 years in the construction workers, the latter accounting for over 70% of the combined sample. Furthermore, the mean age at death was highly variable, ranging from 61 years in the employee sample and 66 years in construction workers to 70+ years in the other cohorts.

At a minimum, the authors could have addressed these problems by other methods ^11, 12^ providing the following information:

• Estimate adjusted hazard ratios for each cohort separately. This provides information about which cohorts are driving the pooled results, and it is especially important when over 70% of the sample was in the construction worker cohort.
  This also addresses the selection problem tied to cohort populations with considerably different age distributions and demographic characteristics. Consider the four cohorts (two population-based and two convenience samples) that enrolled participants at or above a mean age of 50 years. By definition, everyone in these cohorts had already lived at least 50 years and was alive at enrollment. Now compare them with the construction worker cohort, which was enrolled at a mean age of 34 years. Sixteen years later, at age 50, less than 100% of this cohort was still living and contributing person-years, which makes the survivors fundamentally different people compared with those who were enrolled after age 50. The pooled analysis methodology from Byhamre *et al.* ignored this serious problem, and their outcomes may have been significantly biased.
• Estimate a “fixed effects” model by assigning indicator (dummy) variables for cohorts and interacting the cohort variables with age and education. This will allow correlation between the cohorts and snus use and between the cohorts and mortality. Furthermore, the interaction between cohorts and age would allow the effect of age on mortality to differ by cohorts.

## The tables are uninformative

Byhamre *et al.* provided the “Characteristics of included cohorts...” in Table 1 of their publication, and we have used some of this information to illustrate heterogeneity and other problems. ^1^ However, this table does not reflect Byhamre *et al.*’s actual analytic sample (n = 169,103), because it includes the characteristics of all male participants from the eight cohorts (n = 383,025), including ever regular smokers and others that the authors excluded. Simply put, including information on 214,000 participants that aren’t in the analysis is not only uninformative, it is misleading and must be revised.

Although Byhamre *et al.*’s Table 2 provides information on some baseline characteristics in the analytic sample, it presents the pooled characteristics, which is uninformative because it further conceals large differences between the cohorts. For example, variations in enrollment ages across the cohorts have substantial impact on BMI, physical activity, and alcohol use that may overwhelm and obfuscate any differences due to snus use. The authors should have reported the baseline characteristics according to cohorts, and cohorts by snus use.

Furthermore, the authors did not provide adequate definitions for snus use, the sole exposure of interest in the study. Thus, questionnaires containing actual questions about snus use from each cohort is a prerequisite. According to the authors, baseline snus use was not consistent throughout the cohorts. Some studies did not have information on former snus use, while others did not have information on amount and/or duration.

## The exclusion of smokers was not validated and eliminates important information

The authors excluded participants “reporting ever **regular** use of cigarettes” (emphasis added), which was intended “to eliminate potential residual effects”, a vague reference to an unspecified statistical problem. The authors provided no foundation for this choice and no evidence that their objective was met. Furthermore, snus users who were less-than-regular cigarette smokers – those with a smoking history and some dual users – were still in the pooled analytic cohort. ^13^ Less-than-regular smoking is likely because of the lower social acceptability of smoking vis-à-vis snus in Sweden. ^14, 15^

The authors could validate whether their analytic tactic excluded smoking by reporting the results for two disease categories: cancer of the trachea, bronchus, and lung (ICD-10 codes C33–C34) and chronic lower respiratory diseases (J40-J47). If they truly eliminated smokers from their analyses, they should not have found any significantly increased hazard ratios for these diseases among snus users.

Excluding “ever regular” smokers is a routine practice in Swedish follow-up studies of snus use, but it also eliminates a lot of valuable information and may bias the results. First, partial exclusion of an exposure closely associated with snus use precludes assessment of interactions. Second, the findings on snus use in this and similar studies completely lack context with respect to the effects of smoking on mortality. ^16^

## Summary

In conclusion, Byhamre *et al.* ^1^ published an analysis with several flaws that render their results uninformative and possibly misleading. The authors should publish revised tables and re-estimate their analyses as described above to address these problems. These measures are readily achievable, and the validity of the results will be considerably enhanced.

## Data availability

No data is associated with this article.
