# Supplementary material for: Heterogeneity and other problems in a pooled analysis of snus use and mortality
Source: F1000Res. 2021 May 14;10:388. [Version 1] doi: 10.12688/f1000research.52127.1 (PMC8917321; doi:10.12688/f1000research.52127.1)
Supplement: Peer review history

# Peer review: Reviewer response for version 1

**DOI:** 10.5256/f1000research.55362.r124659

**Published:** 2022-03-11

**Authors:** Tord Finne Vedoy

**Affiliations:** 1 Department of Alcohol Drugs and Tobacco, Norwegian Institute of Public Health, Oslo, Norway

The debate on the degree to which snus is associated with increased morbidity or mortality, and how, has been going on for some time.

 Although the discussion may seem most relevant for those relative few who use snus or live in a society where snus use is common, the debate should interest tobacco researchers generally for (at least) two reasons: First, snus is one of the products that has been both a popular and long-lasting harm reducing alternative to cigarette smoking. Correct estimates of the harms associated with snus use are necessary to assess the harm-reducing potential of snus use. Second, estimating the harmfulness of snus use requires that researchers take into account the use of two distinct but related tobacco products. Both these dimensions can inform similar discussions on the harm-reducing potentials of other and more recent nicotine products, for example, e-cigarettes.

 Because few countries have a long history of snus use, there is limited data on individual snus and other tobacco use patterns and health outcomes from national registers. Consequently, many of the studies concerning the association between snus use and registered mortality has been based on the same set of (Swedish) cohorts and examined by the same researchers.

 I find that the issues raised by Rodu and Plurphanswatare are warranted and should be relatively easy to address. A revised study by Byhamre *et al. *would be of great interest to both debates on snus use and public health and snus use and harm reduction.

Are arguments sufficiently supported by evidence from the published literature or by new data and results?

Yes

Is the conclusion balanced and justified on the basis of the presented arguments?

Yes

Is the rationale for commenting on the previous publication clearly described?

Yes

Are any opinions stated well-argued, clear and cogent?

Yes

Reviewer Expertise:

Social explanations of tobacco use and social inequality in tobacco use.

I confirm that I have read this submission and believe that I have an appropriate level of expertise to confirm that it is of an acceptable scientific standard.

# Peer review: Reviewer response for version 1

**DOI:** 10.5256/f1000research.55362.r85432

**Published:** 2021-06-03

**Authors:** Marewa Glover

**Affiliations:** 1 Centre of Research Excellence: Indigenous Sovereignty & Smoking (COREISS), Auckland, New Zealand

The letter 'Heterogeneity and other problems in a pooled analysis of snus use and mortality' raises concerns about the method used in a paper by Byhamre *et al.*, 2021 that was published in the *International Journal of Epidemiology* (Vol.49). The Byhamre *et al. *paper entitled 'Swedish snus use is associated with mortality: a pooled analysis of eight prospective studies' claimed to have found a positive association between snus use and mortality, where previous studies by the same group had not found this association. Rodu and Plurphanswat outline a range of methodological flaws in the Byhamre *et al.* analysis.

 The critique is straightforward and clearly written. The authors have narrowly focused on the poor use of statistical analysis methods. They could have gone further.

 A more in-depth critical and systematic review of the literature being produced by the Swedish Collaboration on Health Effects of Snus is warranted. Particularly, future reviews should include an analysis of Risk of Bias. It is a minor example, but it is indicative that the authors on the Byhamre *et al. *paper do not consider the potential bias inherent in their focus on mostly their own previous work. Nine of the Byhamre *et al.* authors were investigators and authors on one or more of the eight studies the ‘pooled’ data were extracted from. This is a form of authorship bias.

 There is a much larger pool of research that has sought to determine and quantify the risks of snus than indicated by Byhamre *et al.*’s selection. Future critical reviews would need to identify if selection bias is a further limitation of the Byhamre *et al.* analysis.   

 Critiques of published science make an important contribution to science. To ensure knowledge is sound, these types of critiques could even be said to be essential. Seriously flawed studies can mislead policy makers and politicians and lead to decisions about healthcare that could cause harm.

Are arguments sufficiently supported by evidence from the published literature or by new data and results?

Yes

Is the conclusion balanced and justified on the basis of the presented arguments?

Yes

Is the rationale for commenting on the previous publication clearly described?

Yes

Are any opinions stated well-argued, clear and cogent?

Yes

Reviewer Expertise:

Behavioural science; tobacco dependency and cessation; tobacco control; harm reduction; mixed methods from trial design to in-depth qualitative; narrative and systematic reviews.

I confirm that I have read this submission and believe that I have an appropriate level of expertise to confirm that it is of an acceptable scientific standard.
